# Roles of IgE and Histamine in Mast Cell Maturation

**DOI:** 10.3390/cells10082170

**Published:** 2021-08-23

**Authors:** Satoshi Tanaka, Kazuyuki Furuta

**Affiliations:** 1 Department of Pharmacology, Division of Pathological Sciences, Kyoto Pharmaceutical University, Misasagi Nakauchi-cho 5, Yamashina-ku, Kyoto 607-8414, Japan; 2 Department of Immunobiology, Okayama University Graduate School of Medicine, Dentistry and Pharmaceutical Sciences, Tsushima naka 1-1-1, Kita-ku, Okayama 700-8530, Japan; furutak@okayama-u.ac.jp

**Keywords:** mast cell, IgE, histamine, differentiation, allergy, inflammation, MRGPRX2, omalizumab, urticaria

## Abstract

Mast cells are activated upon immunoglobulin E (IgE)-mediated antigen stimulation, and release a wide variety of mediators, including histamine to trigger inflammatory responses. The surface expression levels of Fcε receptor I (FcεRI), a high affinity receptor of IgE, were found to be positively regulated by IgE. IgE could protect murine cultured mast cells from apoptotic cell death induced by the deprivation of interleukin-3 and a certain kind of IgE could activate immature mast cells in the absence of antigens, leading to the release of pro-inflammatory cytokines and a transient increase in histamine synthesis. Histamine synthesis in mast cells was found to be required for the maturation of murine connective tissue-type mast cells, raising the possibility that IgE indirectly modulates local mast cell maturation. Although it remains controversial to what extent this concept of “monomeric IgE effects” could have relevance in the modulation of human mast cell functions, the therapeutic effects of anti-IgE antibodies might be accounted for in terms of the decreased serum IgE concentrations. Because drastic increases in serum IgE concentrations are often observed in patients with atopic dermatitis and chronic urticaria, a close investigation of the roles of IgE in mast cell maturation should contribute to development of novel therapeutic approaches for these inflammatory diseases.

## 1. Introduction

Mast cells are distributed in nearly all vascularized tissues, and are involved in the regulation of a wide array of immune responses, including IgE-dependent immediate allergy [1,2,3]. Early studies suggested, based on the bone marrow transplantation study, that murine tissue mast cells should originate in the hematopoietic stem cells [4]. They also raised the possibility that tissue mast cell populations should be locally maintained. Recent lineage tracing studies of murine mast cell progenitors revealed that yolk sac-derived progenitors should be involved in the maintenance of local mast cell populations, at least in the neonatal stage [5,6]. Although it remains to be clarified which kind of progenitors mainly contribute to the local homeostasis of tissue mast cells in adult mice, inflammation might drastically change the tissue mast cell populations by recruiting bone marrow-derived mast cell progenitors [7,8].

Mast cells are generally identified by their surface expression of c-kit, which is the receptor of the stem cell factor (SCF, c-kit ligand), and FcεRI, which is the high affinity receptor of IgE, and these two receptors are closely associated with the maturation and activation of mast cells. Several murine strains that harbor mutated c-kit genes have been used as mast cell-deficient models [9], indicating the essential role of c-kit in the maturation of tissue mast cells. IgE-bound FcεRI is aggregated in the presence of multivalent antigens, and thereby induces activation of mast cells. Elevated serum concentrations of IgE were often found to be closely associated with a series of allergic diseases, such as atopic dermatitis, allergic asthma, and chronic urticaria, and lowering IgE levels might be beneficial for patients [2]. We focus here on the relationship between IgE and local mast cell maturation, with a particular attention on histamine synthesis in mast cells. Accumulating evidence suggests that elevated levels of IgE should affect the local immune circumstances in mice, and these murine studies might contribute to the development of novel therapeutic approaches of IgE-mediated chronic inflammatory diseases.

## 2. Roles of IgE in Urticaria

**Figure 1:**
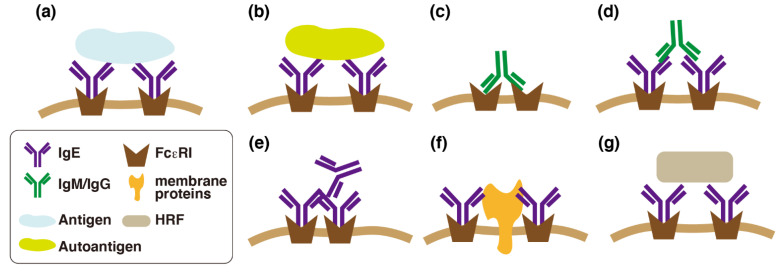
Various modes of Fcε receptor I (FcεRI)-mediated activation of mast cells. (**a**) Mast cells are activated by antigens (allergens) upon immunoglobulin E (IgE)-mediated cross-linking of FcεRI. (**b**) Autoantigens recognized by IgE could cross-link FcεRI. (**c**) Autoreactive IgMs/IgGs that recognize subunits of FcεRI could cross-link FcεRI. (**d**) Autoreactive IgMs/IgGs that recognize IgE could cross-link FcεRI. (**e**) Aggregated IgEs could cross-link FcεRI without the antigens. (**f**) Cis-interaction between IgEs and some glycosylated membrane proteins could cross-link FcεRI. (**g**) HRF could cross-link FcεRI through interaction with specific types of IgE (IgE^+^).

The concept of therapy with an anti-IgE antibody initially targeted the interruption of IgE binding to FcεRI. However, it has been recognized that decreased serum IgE levels and the subsequent downregulation of FcεRI should play critical roles in its therapeutic effects. Serum concentrations of IgE in patients with allergic asthma were found to be significantly decreased when they were treated with omalizumab, a humanized IgG that specifically binds to human IgE [10,11]. Surface expression levels of FcεRI in human basophils were found to be correlated with serum IgE concentrations [12,13]. Elevated levels of serum IgE were often found not only in atopic patients but also in chronic urticaria. Omalizumab is now regarded as the first-line drug for antihistamine-resistant chronic urticaria [14,15,16]. Omalizumab was found to be more effective for chronic urticaria patients with higher serum concentrations of IgE, whereas cyclosporine was efficacious for those with lower IgE [15]. The pathological roles of IgE and FcεRI in chronic urticaria are classified into type I and type IIb; in the type I group, autoreactive IgEs, which could recognize autoantigens, such as thyroid peroxidase, tissue factor, thyroglobulin, double strand DNA, and IL-24, are produced [17,18,19,20], and in the type IIb group, IgM or IgG that could recognize IgE or FcεRI is produced. Because mast cells could undergo degranulation in response to trace amounts of antigens, these findings might account at least in part for the FcεRI-mediated pathological responses in chronic urticaria (Figure 1b–d). However, it remains largely unknown how IgE is involved in exacerbation of chronic urticaria. Interestingly, omalizumab was also found to be effective for patients with normal IgE levels or those without allergen-specific IgE [21,22,23]. It may reflect the presence of trace amounts of IgE being raised against unidentified autoantigens. Further investigation of the role of IgE in the functional changes in tissue mast cells will lead to a better understanding of the pathophysiological roles of tissue mast cells.

## 3. Effects of IgE in the Absence of Antigens

The high affinity binding of IgE to FcεRI and its slow dissociation indicate that tissue mast cells should be consistently armed with IgE [24]. Murine perivascular cutaneous mast cells were found to actively capture IgE, by extending the cell processes across the vessel wall [25]. Little attention has been paid to the changes of mast cells that occur through binding of IgE to FcεRI, although a large part of cultured mast cell models have vacant FcεRI on the surface.

### 3.1. Effects of IgE in the Absence of Antigens (Monomeric IgE Responses)

Early studies indicated a positive correlation between extracellular IgE levels and IgE binding to FcεRI, indicating the presence of a kind of positive feedback loop [26,27,28,29]. IgE-mediated upregulation of FcεRI was later found to be the result of surface stabilization of FcεRI in the presence of higher concentrations of IgE [30,31]. In 2001, two groups simultaneously demonstrated that IgE could interfere with apoptotic cell death induced by growth factor deprivation using IL-3-dependent murine bone marrow-derived cultured mast cells (BMMC) [32,33]. Although both studies found the anti-apoptotic effects of IgE, one group alone showed significant amounts of cytokine production, such as TNF-α, IL-4, IL-6, and IL-13 [33]. This discrepancy was partially resolved by the following study, in which the potentials of cytokine induction were found to be varied among individual IgE clones [34]. They classified IgEs into two categories, highly cytokinergic IgEs (HC-IgEs), which could induce massive cytokine releases in the absence of antigens, and poorly cytokinergic IgEs (PC-IgEs), which have little or no such capacity. HC-IgEs were found to induce a wide variety of responses in addition to anti-apoptotic cell death, such as histamine synthesis, adhesion to fibronectin, migration, and degranulation, in BMMCs and rat basophilic leukemia, RBL-2H3 [35,36,37,38,39]. One of the most potent HC-IgEs was the clone SPE-7, which was raised against the dinitrophenyl (DNP) group and has been utilized as one of the most typical model systems for IgE-mediated antigen stimulation together with its antigen, DNP-conjugated albumin. Differences in the potential for cytokine induction among various IgE clones suggested that Fab regions of IgE should be involved in triggering the IgE-mediated activation of mast cells. Inhibition by a monovalent hapten, DNP-lysine, supported this hypothesis [40,41]. HC-IgE-mediated resistance to apoptotic cell death induced by IL-3 deprivation was found to result from the autocrine effects of IL-3 produced by BMMCs in the presence of IgE [42]. Early findings that PC-IgEs did not induce the release of survival factors, such as IL-3 and c-kit ligand [32], raised another possibility that the intracellular signaling pathway downstream of FcεRI should be directly involved in the survival.

Sakanaka et al. made a comparison of the sensitizing effects between two IgE clones, SPE-7 (HC-IgE) and IgE-3 (PC-IgE) [43]. When BMMCs were sensitized with SPE-7, antigen-induced responses, such as degranulation and the production of IL-6 and TNF-α, were significantly down-modulated in an IgE concentration-dependent manner. No such responses were found in the cells sensitized with IgE-3. This down-modulation was accompanied by the decreased phosphorylation of Syk. SP600125, a specific inhibitor of JNK, was found to restore the antigen-induced responses in BMMCs sensitized with SPE-7. These findings suggest that SPE-7-induced phosphorylation of JNK is involved in the attenuated responses upon antigen stimulation.

### 3.2. Possible Molecular Mechanisms of IgE-Mediated FcεRI Activation

Because the effects of IgE in the absence of antigens were observed in the buffer solutions containing bovine serum albumin, the possibility that the constituents in the fetal bovine serum could cross-link the FcεRI by binding to the specific IgE clone might be excluded. James et al. demonstrated, using the approach of structural biology, that SPE-7 should have a potential to bind two or more structurally different antigens [44]. This finding could lead to the hypothesis that a weak but significant interaction between the IgE molecules mediate the effects of HC-IgEs, in particular in the presence of high concentrations of IgE. Recently, a detailed characterization of SPE-7 was performed. They demonstrated that a soluble free form of SPE-7 could interact with Fab regions of the FcεRI-bound form, and this interaction triggers2 the activation of cultured mast cells [45] (Figure 1e). They further reported that commercially available SPE-7 contains trace amounts of the dissociation-resistant IgE trimer, and that a highly purified form of SPE-7 could no longer trigger mast cell activation in the absence of antigens [46], although the possibility of contamination of aggregated forms of IgE was excluded based on the results of liquid chromatography and ultracentrifugation in early studies. Because the activation of FcεRI by its cross-linking is a very sensitive reaction, it might be plausible that a weak interaction induced by trace amounts of the IgE complex, such as IgE trimers, is involved.

Shade et al. recently reported that the sialylation of IgE plays a critical role in FcεRI-mediated activation of mast cells [47]. They demonstrated that the removal of sialic acid of IgE resulted in the drastic attenuation of FcεRI-mediated activation of human and murine mast cells. It might be intriguing to investigate the relationship between the heterogeneity of the *N*-glycan composition and the potency as HC-IgEs. Because removal of sialic acid leads to the exposure of galactose at the end of *N*-glycans, galactose-binding lectins, such as galectin-3 and galectin-9, may be involved in the suppression of FcεRI-mediated activation of mast cells. Both galectins were found to suppress FcεRI-mediated activation of mast cells, although a previous study using gene-targeted mice that were lacking galectin-3 suggested that galectin-3 enhances the activation [48,49,50]. It is also plausible that unidentified glycosylated membrane proteins are involved in FcεRI-mediated activation (Figure 1f).

The Fab region-mediated activation has also been explored from different points of view. Histamine-releasing factor (HRF) was identified as a secretagogue of human basophils, and its action was found to be mediated by a certain type of IgE (IgE+) [51] (Figure 1g). Because the actions of HRF are mediated by limited populations of IgE, HC-IgEs appear similar to IgE+. However, human IgE+ might not be equivalent to HC-IgEs, because human IgE alone could not directly activate human cultured mast cells in a similar fashion to murine cultured mast cells [52]. The close investigation of the molecular identity of IgE indicated that the Fab region should be involved in HRF-mediated degranulation of mast cells and basophils [53]. Because HRF was found to be identical to the translationally-controlled tumor protein that is required for cell cycle progression, it might be difficult to clarify the pathophysiological roles of HRF using gene-targeted mice lacking HRF. However, suppression of extracellular functions of HRF might be a novel therapeutic approach for allergic diseases.

### 3.3. Differences between Monomeric IgE Responses and IgE-Mediated Antigen Stimulation

**Figure 2:**
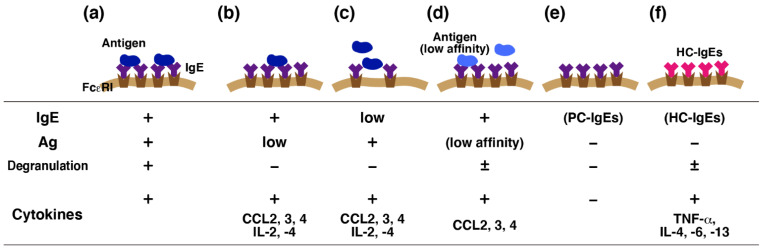
Finely tuned Fcε receptor I (FcεRI)-mediated activation of mast cells. Immunoglobulin E (IgE)-mediated antigen stimulation triggers both degranulation and release of various cytokines (**a**). Accumulating evidence suggests that different sets of mediators should be released from activated mast cells without degranulation. Early studies indicated that several chemokines and cytokines are released from mast cells upon weak antigen (Ag) stimulation without degranulation (**b**,**c**). Such preference was also observed in mast cells activated by antigens with low affinity to IgE (**d**). Poorly cytokinergic (PC)-IgEs were found only to prevent apoptosis of mast cells, whereas highly cytokinergic (HC)-IgEs were found to induce a massive cytokine release (**e**,**f**). This variation may affect the local immune responses, such as the leukocyte recruitment.

A series of studies about IgE-mediated activation of cultured mast cells demonstrated that high concentrations of HC-IgEs (1–5 µg/mL) could induce degranulation [37,38,43,45,46]. Huber et al. previously reported that SPE-7 alone could induce significant levels of cytosolic Ca^2+^ mobilization and degranulation in the cultured mast cells, prepared from bone marrow of gene-targeted mice lacking Src homology 2-containing inositol phosphatase (SHIP) [54]. They suggested that IgE alone has the potential to induce mast cell activation, including degranulation, and SHIP could suppress activation by IgE alone as a gatekeeper. SPE-7 alone did not induce degranulation in the wild type mice, although they used it at a high concentration (10 µg/mL). It might be intriguing to investigate how HC-IgEs at lower concentrations could induce massive cytokine secretion without degranulation. HC-IgEs induced a series of events observed upon IgE-mediated antigen stimulation, such as phosphorylation of MAPK and Ca^2+^ mobilization, and HC-IgE-mediated responses depended on Lyn, Syk, and phospholipase C [33,34,35,36,37,38,39,40,41], although degranulation occurred only in the presence of higher concentrations of HC-IgEs. Weak but prolonged phosphorylation of ERK and sustained Ca^2+^ mobilization was found to be characteristic of IgE-triggered responses in the absence of antigens [33,41,55]. HC-IgE-mediated Ca^2+^ mobilization was resistant to La^3+^ and Gd^3+^, which could suppress Ca^2+^ influx induced upon IgE-mediated antigen stimulation [41,56]. Accumulating studies indicated that Ca^2+^ influx induced upon IgE-mediated antigen stimulation is mediated mainly by the STIM1-Orai1 system [57]. It remains to be clarified which kind of Ca^2+^ channels mediate HC-IgE triggered Ca^2+^ influx, although several transient receptor potential families were found to be expressed in mast cells [58]. Distinct sensitivity to La^3+^ and Gd^3+^ indicated that the molecular mechanisms of Ca^2+^ influx are different between stimulation with HC-IgEs and antigen stimulation. HC-IgE-induced activations of mast cells were found to be significantly suppressed by several protein kinase inhibitors, such as Gö6976, which could suppress conventional protein kinase C (PKC) [35,41]. Liu et al. demonstrated using a mouse mastocytoma, P-815, which lacks the expression of FcεRI, that the reconstitution of FcεRI made the cells sensitive to IgE-mediated antigen stimulation but not to SPE-7 alone [56]. P-815 was found to lack the expression of PKCβII, of which the expression is shared by various murine cultured mast cells, and the reconstitution of PKCβII restored IgE-triggered Ca^2+^ mobilization in the absence of antigens. These findings suggested that PKCβII is dispensable for antigen-induced Ca^2+^ influx, but essential for IgE-triggered Ca^2+^ mobilization. Gonzalez-Espinosa et al. demonstrated that IL-2, IL-4, and several chemokines, such as CCL2, CCL3, and CCL4, were transcriptionally induced in IgE-sensitized BMMCs in the presence of low concentrations of antigen, where no detectable levels of degranulation were found, whereas IL-3, IL-6, and IL-13 were induced only in the presence of high concentrations of the antigen, indicating that weak stimulation of FcεRI could induce cytokine/chemokine induction without degranulation [59]. Suzuki et al. extended this concept and demonstrated that one of Src family kinases, Fgr, is involved in the activation of mast cells induced by low affinity antigens [60]. These findings suggest that FcεRI-mediated activation of mast cells are precisely regulated in response to a wide variety of environmental conditions (Figure 2). However, because significant releases of IL-3, IL-6, and IL-13 were found in BMMCs stimulated with HC-IgEs alone without degranulation, the effects of HC-IgEs were quite different from those observed upon weak antigen stimulation. Further analyses are required to fully clarify the mechanism of action of HC-IgEs.

### 3.4. Antigen-Independent Roles of IgE in Cutaneous Mast Cells

Bryce et al. demonstrated that the presence of IgE is required for hapten-induced contact sensitivity in gene-targeted mice lacking IgE [61]. Impaired contact sensitivity responses in the IgE^−/−^ mice were restored by intravenous injection before hapten sensitization with monoclonal IgE that was unrelated to the hapten. One of the mast cell-specific granule proteases, mouse mast cell protease-6 (MMCP-6), was transcriptionally downregulated in the cutaneous tissues of the IgE^−/−^ mice, but was upregulated by the injection of IgE. These findings strongly suggested that IgE could enhance the potential to trigger inflammatory responses in cutaneous mast cells in an antigen specificity-independent fashion. Because the surface FcεRI in the cutaneous mast cells might be occupied by IgE in the wild type mice, these findings were obtained under the artificial settings. However, deprivation of circulating IgE by an anti-IgE antibody may impair the functions of newly recruited tissue mast cells.

## 4. Histamine Synthesis in Mast Cells

Mast cells produce a wide variety of proinflammatory mediators upon various stimuli [2,3]. Among them, histamine plays a critical role in triggering allergic and inflammatory responses. In 1953, Riley and West first identified the presence of histamine in mast cells [62]. Histamine is synthesized through the decarboxylation of _L_-histidine, which is mediated by histidine decarboxylase (HDC) [63]. A variety of pathophysiological functions of histamine are mediated by four histamine receptor subtypes, H_1_, H_2_, H_3_, and H_4_ [64]. Accumulating evidence indicates that allergic responses induced by histamine are mediated mainly by the H_1_ subtype [65]. Many H_1_ receptor antagonists have been developed as antiallergic pharmaceuticals, antihistamines, which are also used as the first-line drug for urticaria. Recent studies suggest that the blockade of H_4_ receptors is also promising for inflammatory diseases, such as asthma and pruritis [66]. The H_4_ subtype was found to be preferentially expressed in leukocytes, and mediates histamine-induced chemotaxis [67]. Ohsawa et al. reported that the combination of an H_4_ receptor antagonist, JNJ7777120, and an H_1_ receptor antagonist, olopatadine, showed an excellent therapeutic efficiency in the picryl chloride-induced chronic dermatitis model [68].

### 4.1. The Rate-Limiting Enzyme for Histamine Synthesis: Histidine Decarboxylase

HDC is the rate-limiting enzyme for histamine synthesis. It belongs to the family of vitamin B_6_-dependent decarboxylases, including aromatic-L-amino-acid decarboxylase (dopa decarboxylase). HDC is different from the other decarboxylases in terms of the presence of the carboxyl-terminal extra domain. The cDNA of HDC encodes a protein, of which the molecular weight is 74-kDa, whereas the purified enzyme has been reported to consist of a homodimer of the 53-kDa subunit [69]. The 74-kDa form of HDC localized in the cytosol was found to undergo ubiquitination and proteasomal degradation [70]. Post-translational processing of HDC was found to be mediated by caspase-9 in a mouse mastocytoma cell line, P-815, and resulted in the increase in enzymatic activity [71]. In a rat basophilic leukemia cell line, RBL-2H3, the 74-kDa form, was found to be localized in the cytosol, whereas the 53-kDa form was in the granules upon density gradient fractionation [72]. These findings strongly suggest that histamine synthesis occurs in two spatially distinct compartments, cytosol and granules, in mast cells and basophils, although it remains to be clarified how the 53-kDa form could be localized to the granules. In murine neutrophils, HDC was rapidly processed and the resultant 53-kDa form was also localized in the granules [73].

HDC was found to be transcriptionally induced upon various stimuli [69]. The drastic induction of histamine synthesis (~200-fold) was found in BMMCs, which is regarded as an immature murine mast cell model, when they were treated with an HC-IgE, SPE-7 [41]. BMMCs were found to be sensitive to Ca^2+^-mobilizing reagents, such as thapsigargin and a calcium ionophore, A23187, which could also drastically induce histamine synthesis. Only the 74-kDa form of HDC was detected in BMMCs treated with SPE-7, indicating that IgE alone induces histamine synthesis mainly in the cytosol. The finding that only a moderate increase in histamine synthesis was found in rat purified peritoneal mast cells stimulated with SPE-7 [41] raises the possibility that sensitivity to HC-IgEs is specific to immature mast cells.

### 4.2. Gene Targeting of Hdc

Ohtsu et al. first reported the gene targeting of mice lacking HDC [74]. De novo histamine synthesis was abolished in this *Hdc*^−/−^ strain, but trace amounts of histamine, which originated in the standard diet, might be detected in several tissues. Unexpectedly, mast cells in the cutaneous tissues and peritoneum of the *Hdc*^−/−^ mice exhibited aberrant granule morphology. Transmission electron microscopy revealed that they had granules with extremely low density. The protein expression levels of MMCP-4, MMCP-5, and carboxypeptidase A were decreased in the *Hdc*^−/−^ mast cells. The *Hdc*^−/−^ mice were resistant to IgE-mediated passive cutaneous responses and compound 48/80-induced cutaneous extravasation, and dietary supplemented histamine could partially restore these responses [75]. These findings consolidated that histamine is one of the primary inflammatory mediators, although it remains to be clarified how impaired granule formation in mast cells are involved in the decreased inflammatory responses in the *Hdc*^−/−^ mice. Conditional deletion of HDC in neutrophils raised the possibility that neutrophil-derived histamine may partially contribute to IgE-mediated systemic anaphylaxis [76].

Yang et al. found that the *Hdc*^−/−^ mice are more susceptible to chemical carcinogenesis [77]. They identified that the HDC^+^ cells infiltrated in the tumor tissues as CD11b^+^Ly6G^+^ immature myeloid cells, which supported the tumor growth. Exogenously added histamine promoted the maturation of the *Hdc*^−/−^ CD11b^+^Ly6G^+^ immature myeloid cells, which resulted in the loss of their immunosuppression. Recently, Takai et al. demonstrated, through using a bacterial artificial chromosome DNA-directed GFP reporter transgenic mouse model, that CD11b^+^Ly6G^+^Ly6C^low^ myeloid cell population accumulated in the lung during sepsis [78]. It remains to be clarified how histamine is involved in the maturation of CD11b^+^Ly6G^+^ cells.

## 5. Histamine-Mediated Granule Maturation of Mast Cells

Mast cells with aberrant granules were observed in the *Hdc*^−/−^ mice, although it remained unknown how histamine is involved in granule formation. Previous studies using the gene-targeted mice lacking heparan sulphate *N*-deacetylase/*N*-sulphotransferase 2 (NDST2) demonstrated that a lack of heparan sulphate caused a drastic decrease in the amount of granule proteases and histamine [79,80]. Because no significant changes were found in mRNA expression of granule proteases, they suggested that impaired electrostatic interaction between heparan sulphate and granule proteases leads to instability of the granule components in the *Ndst2*^−/−^ mast cells. A comparison of gene expression profiles of peritoneal mast cells revealed that a series of genes involved in granule formation, such as *Ext1*, *Ndst2*, *Mcpt4*, *Mcpt5*, *Mcpt6*, *Mcpt7*, and *Ctsg*, were significantly downregulated in the *Hdc*^−/−^ mice, whereas that of *Hdc*^−/−^ BMMCs were unchanged [81]. Co-culture of BMMCs with Swiss 3T3 cells in the presence of c-kit ligand was found to reflect at least partially the process of maturation of connective tissue-recruited immature mast cells [82,83]. This model with the *Hdc*^−/−^ BMMCs reproduced the impaired granule formation observed in the *Hdc*^−/−^ mice [81]. Exogenously added histamine increased the frequency of Safranin O-positive cells and chymotryptic activity, indicating that histamine could enhance at least partially granule formation. Accumulating evidence suggested that the electrostatic interaction among the granule components is critical for the stability of granule proteases [84], and that histamine interacts with heparan [85]. However, although inhibition of vesicular monoamine transporter-2 by tetrabenazine nearly completely suppressed the granule storage of histamine in the wild type co-cultured mast cells, the number of Safranin O-positive mature mast cells was unchanged [81], indicating that the absence of granule storage of histamine should not affect the granule formation. Although the positive effects of histamine were reproduced by clobenpropit and VUF8430, indicating the possible contribution of the H_4_ receptor subtype, a similar aberrant granule formation to that of the *Hdc*^−/−^ mast cells was not observed in the *Hrh4*^−/−^ mast cells. Reconstitution analyses, in which BMMCs were injected into the peritoneal cavity of mast cell-deficient *Kit*^W^/*Kit*^W-V^ mice, indicated that granule maturation is dependent solely on the presence of HDC in BMMCs. These findings strongly suggested that endogenous histamine synthesis is required for intact granule formation [81].

These findings might exclude the possibility that receptor-mediated actions or granular localization enhance histamine-induced granule maturation. Schneider et al. demonstrated that organic cation transporter-3 (OCT-3) mediates the bidirectional transport of histamine and regulates the cytosolic histamine levels [86]. Exogenously added histamine was found to suppress the production of IL-4, IL-6, IL-13, and endogenous histamine synthesis in murine basophils stimulated with IL-3. Their findings suggested that the inhibitory effects of exogeneous histamine is mediated by histamine incorporated into the cytosol through OCT-3. Fesus et al. demonstrated an increase in the enzymatic activity of transglutaminase and in the amount of protein-bound γ-glutamylhistamine (histaminylation) in a murine mast cell line, activated by the antigen or a Ca^2+^ ionophore [87]. Transglutaminases catalyze the formation of an amide bond between the γ-carboxamide group of glutamine residues and the primary amino groups including ε-amino group of lysine in a Ca^2+^-dependent manner [88]. Vowinckel et al. demonstrated that G proteins, such as Cdc42, G_αo1_, and G_αq_, undergo a post-translational modification, in which the glutamine residues are covalently modified by histamine in P-815 cells [89]. They observed the expression of transglutaminase-1, -2, and -3 in P-815 cells, and this indicated that transglutaminase-2 is involved in this response. In vitro analyses indicated that histaminylation of G proteins result in the decrease in GTPase activity, leading to prolonged activation of the modified G proteins.

Histamine contents in BMMCs derived from female C57BL/6J mice were found to be higher than those from male mice [90]. Histamine contents in BMMCs were significantly decreased when they were prepared from the adult female mice exposed to androgen at their perinatal stages. Under this condition, the expression of MMCP-4 and carboxypeptidase A3, and the content of serotonin were significantly decreased. It remains to be clarified how histamine synthesis was decreased in BMMCs derived from the androgenized female mice.

## 6. Pro-Inflammatory Roles of Granule Components

Accumulating evidence indicates that mast cells acquire the potential to produce a specific set of inflammatory mediators in response to changes in the microenvironment. During the process of differentiation into connective tissue-type mast cells, the amount of a large part of granule components were found to be increased. Recent studies demonstrated that granule components are involved in a wide variety of inflammatory responses.

Although mast cells were found to undergo degranulation in response to IgE-independent stimuli, it remains unknown how such secretagogues induce degranulation. Recent progress has revealed that Mas-related G protein-coupled receptor (Mrgpr) family accounts for a substantial portion of IgE-independent degranulation. Mrgpr gene family might be a novel therapeutic target of inflammatory diseases, such as chronic urticaria and atopic dermatitis [91,92,93].

### 6.1. Pathophysiological Role of Granule Components

Mast cell granules share many characteristics with lysosomes. The activity of a lysosomal enzyme, β-hexosaminidase, is measured to determine the percentages of degranulation, instead of measuring the amount of histamine.

The expression profiles of granule proteases were found to reflect the heterogeneity of tissue mast cells. Granule proteases are classified into three categories, tryptase, chymase, and carboxypeptidase A3. Human mast cells are categorized into MC_TC_, which expresses tryptase and chymase; and MC_T_, which expresses only tryptase. Murine mast cells could be categorized into connective tissue-type mast cells (CTMCs), a majority of which expresses MMCP-4, -5, -6, and -7; and mucosal mast cells (MMCs), which expresses MMCP-1, and -2. Because tryptase was found to activate proteinase activated receptor 2 (PAR-2) [94,95], some of the actions of tryptase might be mediated by PAR-2. Hamilton et al. demonstrated, using gene-targeted mice lacking MMCP-6 and -7, that MMCP-6 and not MMCP-7 are involved in chemically induced colitis [96]. Cui et al. reported that MMCP-6 plays a critical role in an ovalbumin-induced airway hypersensitivity model [97]. The pathological role of MMCP-4 might be complex. MMCP-4 was found to enhance the inflammatory responses in the bleomycin-induced lung inflammation model [98], whereas it had a protective role in airway allergic responses induced by house dust mite through the degradation of IL-33 [99,100]. Mast cells were found to be involved in the limitation of the toxicity of endothelin through the proteolytic activity of carboxypeptidase A3 [101,102]. The sctivation of an endothelin receptor subtype, ET_A_, resulted in degranulation of mast cells, leading to the release of carboxypeptidase A3.

The increased granule storage of heparin is the hallmark of mature connective tissue-type mast cells. Oschatz et al. demonstrated that factor XII-mediated formation of bradykinin was triggered in the presence of heparin, and that bradykinin increased vascular permeability by acting on the kinin B2 receptors, raising the possibility that heparin should have pro-inflammatory roles in addition to the maintenance of granule homeostasis [103].

### 6.2. IgE-Independent Degranulation

Mast cells have a wide variety of surface receptors, and a potential to respond to a diverse array of environmental changes in addition to IgE-mediated antigen stimulation [104]. Among them, polybasic compounds, such as compound 48/80, and a certain kind of bioactive peptides, such as substance P, neurotensin, and anaphylatoxins (fragments of complements, C3a and C5a), were found to directly trigger degranulation of mast cells [105]. These responses were pertussis toxin-sensitive and confirmed only in the connective tissue-type mast cells [106]. Tatemoto et al. first suggested that a member of Mas-related G protein coupled receptors, MRGPRX2, was expressed in human mast cells and is responsible for degranulation induced by mast cell secretagogues, such as mast cell-degranulating peptide, pituitary adenylate cyclase-activating polypeptide, and substance P [107]. Accumulating evidence suggests that possible MRGPRX2 agonists contain a diverse array of endogenous biological mediators, such as neuropeptides and bactericidal peptides, and therapeutic compounds with pseudo allergic effects, such as opioids, vancomycin, quinolone antibiotics, and neuromuscular blocking drugs [91,92,93]. Fujisawa et al. demonstrated that MRGPRX2 was upregulated in cutaneous mast cells of patients with severe chronic urticaria [108]. Mrgpr gene family was originally identified as the genes encoding G protein-coupled receptors expressed in nociceptive sensory neurons [109]. Mrgpr genes are extremely expanded in mice, and it might be difficult to determine the murine counterpart of MRPRX2 [110]. However, McNeil et al., using the gene-targeted mice lacking MrgprB2, demonstrated that the activation of mast cells induced by various MRGPRX2 agonists, such as compound 48/80, substance P, icatibant, ciprofloxacin, and tubocurarine, was totally suppressed in mast cells lacking Mrgprb2 [111]. Yamada et al. found that the mRNA of other Mrgpr subtypes, such as MrgprA4, B1, B10, C11, and E, were expressed in addition to MrgprB2 in murine bone marrow-derived cultured mast cells [112]. The expression of these Mrgpr subtypes was suppressed when the cells were treated with dexamethasone.

It remains to be fully clarified how chronic cutaneous inflammatory diseases, such as chronic urticaria and atopic dermatitis, are exacerbated. Because MRGPRX2 could be activated by various structurally different agonists, it is plausible that MRGPRX2 plays a critical role in mast cell-dependent chronic cutaneous inflammation [93]. Serhan et al. demonstrated, using several gene-targeted mice, that a house dust mite allergen triggered the release of substance P, which activated cutaneous mast cells by acting on Mrgprb2 [113]. Granule maturation of cutaneous mast cells may impact MRGPRX2-mediated inflammatory responses.

## 7. Concluding Remarks

**Figure 3:**
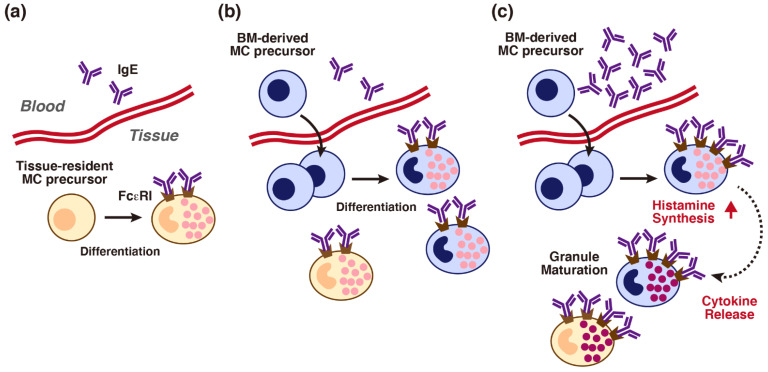
Possible effects of immunoglobulin E (IgE) on local maturation of tissue mast cells. (**a**) Accumulating evidence suggests that local tissue mast cell populations are maintained through the differentiation of the tissue-resident mast cell (MC) precursors. (**b**) Bone marrow-derived MC precursors are infiltrated from the circulation upon inflammation. Such MC precursors might be differentiated in response to the environmental factors. (**c**) Higher concentrations of IgE might induce up-regulation of surface Fcε receptor I (FcεRI) and monomeric IgE effects on infiltrated immature mast cells. An IgE-induced transient increase in histamine synthesis and autocrine/paracrine effects of released cytokines may enhance granule maturation of immature mast cells (dark colored granules).

Accumulating evidence suggests that mast cell-committed progenitors are infiltrated from bone marrow into tissues upon inflammation, and that they then proliferate in response to environmental factors including c-kit ligand. The process of mast cell maturation in the murine connective tissues could be depicted as follows (Figure 3). Surface expression of FcεRI might be induced during maturation, and it is drastically upregulated when immature mast cells are exposed to higher concentrations of IgE because surface expression levels of FcεRI are positively regulated by IgE. Sensitization with a certain type of IgE (HC-IgEs), furthermore, may induce a transient but massive increase in histamine synthesis in tissue mast cells, which is accompanied by the production of several cytokines. Although the molecular mechanism remains to be clarified, it is likely that an increase in cytosolic histamine should promote granule maturation of murine immature mast cells. A rapid and transient increase in cytosolic Ca^2+^ concentrations induced by IgE could lead to transglutaminase-mediated histaminylation of G proteins, which may enhance granule-related gene expression. Gu et al. previously suggested that Rac2 is involved in the maturation of BMMCs [114]. However, it remains to be confirmed whether this scheme is applicable to human mast cell maturation and the exacerbation of chronic inflammatory diseases characterized by increased IgE levels. The human counterpart of HC-IgEs remains to be precisely identified. Furthermore, it remains to be determined whether tissue histamine levels are correlated with serum IgE concentrations.

More attention has been recently paid to the pathophysiological roles of tissue-resident mature mast cells. Previous studies focused mainly on the process of mast cell activation upon IgE-mediated antigen stimulation, using various mast cell lines and immature primary cultured mast cells. This is of great significance to understand the role of mast cells in immediate allergic responses, but is not always applicable to understanding of the pathophysiological roles of local mast cells. The absence of suitable culture models has introduced a delay in the investigation of mature mast cell-related functions, including MRGPRX2-mediated activation. Recent progress in the techniques of mast cell research, such as novel culture systems and gene-targeted mice models, has enabled us to investigate the roles of local mast cell populations. Close investigation of local mast cells will provide us with novel therapeutic approaches for chronic inflammatory diseases.

## Data Availability

Data sharing is not applicable to this article.
